# Unveiling Koro’s diverse conceptualizations across cultures

**DOI:** 10.1192/j.eurpsy.2024.561

**Published:** 2024-08-27

**Authors:** D. Seabra, I. Lopes, J. Moura, J. Leal, T. Rocha, J. Cunha, S. Torres, D. Santos, G. Santos, N. Ramalho

**Affiliations:** ^1^Psychiatry and Mental Health, Centro Hospitalar Barreiro Montijo, Barreiro, Portugal

## Abstract

**Introduction:**

Koro, also known in Cantonese as *Shook Yang*, which literally translates to “shrinking penis”, has its roots in a cultural belief that a mythological figure would steal the penis of his victims. Predominantly reported in Southeast Asia, it involves an acute fear of genital retraction, often accompanied by the belief that this retraction may lead to death. Over the last two centuries, Koro has undergone several attempts to establish its definition and classification, without a true consensus having been reached.

**Objectives:**

This study aims to explore the cultural nuances surrounding Koro and reflect on the various conceptualizations that modulated its definition and nosological classification, from Ancient China until the present.

**Methods:**

A non-systematic literature review with the keywords “koro” and “culture” was conducted.

**Results:**

Koro was only introduced to the Western world during colonial expansion, drawing the attention of several psychiatrists who, in Asian territory, reported numerous cases in natives, making the very first attempts at a nosological classification, whether as an anxiety neurosis, or as an obsessive-compulsive disorder. The literature reveals significant cultural variations in the manifestation of Koro, challenging the traditional psychiatric understanding rooted in Western diagnostic categories. Cultural factors, including societal beliefs, religious practices, and regional variations, emerged as influential contributors to the prevalence and presentation of Koro. Additionally, the study identified instances of Koro evolving in response to cultural shifts and globalization, emphasizing the dynamic nature of this syndrome.

**Conclusions:**

This review underscores the need for a comprehensive understanding of Koro that acknowledges its diverse conceptualizations across cultures. Its occurrence, not only in multiple parts of the world, but also in close relation with various comorbidities, has contributed to the dissolution of its primary identity as a culture-bound syndrome, turning Koro into a moving target.

**Disclosure of Interest:**

None Declared

